# Modified Peroral Endoscopic Myotomy Technique for Type II Achalasia: A Multicenter Retrospective Study

**DOI:** 10.1155/2022/3424470

**Published:** 2022-03-25

**Authors:** Huahui Zhang, Kuangjing Wang, Ying Fang, Zhe Xiong, Min Lin, Lifeng Jiang, Qiuya Niu, Jin Huang

**Affiliations:** ^1^Graduate School of Dalian Medical University, Dalian, China; ^2^Department of Gastroenterology, The People's Hospital of Ma'anshan, Ma'anshan, China; ^3^Department of Gastroenterology, The Affiliated Changzhou No. 2 People's Hospital of Nanjing Medical University, Changzhou, China

## Abstract

**Aim:**

This retrospective study is aimed at evaluating the outcomes of a modified peroral endoscopic myotomy (POEM) technique in patients with type II achalasia.

**Methods:**

We performed a modified POEM procedure, which involved a shorter (total myotomy length = 4 cm), full-thickness myotomy, on 31 patients with type II achalasia. Clinical success rates, technical success rates, pre- and postoperative esophageal manometry results, complications, and reflux-related adverse events were evaluated.

**Results:**

The clinical success (Eckardt score ≤ 3) rates were 100% and 88.9% within 2 years and beyond 2 years postoperatively, respectively. The median lower esophageal sphincter pressures (LESP) decreased from 31.6 (26.7-49.7) mmHg preoperatively to 13.4 (10.5-21.6) and 11.8 (7.4-16.7) mmHg (*P* < 0.001) at 6 and 12 months postoperatively, respectively. The median integrated relaxation pressure (IRP) decreased from 27.8 (20.6-37.5) mmHg preoperatively to 12.9 (11.3-23.4) and 11.6 (9.6-16.8) mmHg (*P* < 0.001) at 6 and 12 months after POEM, respectively. Only one case (3.2%) of mucosal injury, four (12.9%) cases of reflux esophagitis, and two (6.5%) cases of gastroesophageal reflux symptoms were reported.

**Conclusions:**

The modified POEM technique showed excellent outcomes in patients with type II achalasia.

## 1. Introduction

Achalasia is an esophageal motility disorder characterized by incomplete relaxation of the lower esophageal sphincter (LES) and disordered peristalsis in the esophageal body, which induce changes in the esophageal function of bolus transport and food stasis [1]. According to the Chicago classification version 4, achalasia is defined as an abnormal median IRP with 100% failed peristalsis, with three characteristic phenotypes: type I, peristalsis absent; type II, ≥20% swallows with panesophageal pressurization; and type III, ≥20% of swallows with premature contraction [2]. The treatment of achalasia requires lowering the LESP through medications, endoscopic injection of botulinum toxin, pneumatic dilation (PD), or laparoscopic Heller myotomy (LHM). POEM is a minimally invasive endoscopic treatment for achalasia first described by Inoue et al. in 2008 [3]. Since its introduction, thousands of POEM procedures have been performed; POEM has been reported to be safe and effective. Existing uncontrolled reports suggest efficacy equal to or superior to LHM, and emerging randomized controlled trial data suggest that POEM is more effective than PD [4].

There is no consensus regarding the dissection of the sphincter muscles and the overall technique, and even the periprocedural management varies across centers and endoscopists. Most endoscopists selectively dissect only the circular muscle; however, others prefer dissecting both circular and longitudinal muscle layers, but there are no criteria for dissection of the muscle layers.

At our centers, we developed a strategy for treating type II achalasia using high-resolution manometry (HRM) data, in which a shorter tunnel was created and full-thickness dissection of the LES and cardiac sphincter was performed. This study presents an introduction to our procedure, its clinical success, and the rate of adverse events.

## 2. Methods

### 2.1. Patients

Patients with type II achalasia who underwent modified POEM at the Affiliated Changzhou No. 2 People's Hospital of Nanjing Medical University and the People's Hospital of Ma'anshan from January 2015 to August 2020 were enrolled in our study. The inclusion criteria for our study were as follows: (1) patients diagnosed with type II achalasia by clinical symptoms, barium meal, HRM, and esophagogastroduodenoscopy (EGD); (2) age ≥ 18 years; and (3) Eckardt score > 3. The following exclusion criteria were applied: (1) history of gastrointestinal tumors, (2) history of treatment by POEM, (3) history of esophageal or mediastinal surgery, and (4) length of the LES > 4 cm. Finally, 31 consecutive patients were included in this study. All patients had no contraindications for POEM and provided written informed consent before POEM. This study was approved by the Ethics Committee of the Affiliated Changzhou No. 2 People's Hospital of Nanjing Medical University.

### 2.2. Preoperative Evaluation

The patients were examined for symptoms and analyzed using the Eckardt score. They next underwent barium esophagography for esophageal dynamics analysis. HRM was used to characterize the esophageal disorders and measure the length of the LES, IRP, and LESP.

### 2.3. POEM Technique

**Figure 1:**
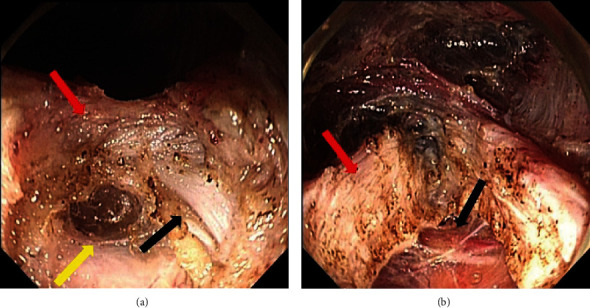
(a) The red arrow indicates the circular muscle layer, the black arrow indicates the longitudinal muscle layer, and the yellow arrow indicates the external coat of the esophagus. (b) The red arrow indicates the circular and longitudinal muscle layers, and the black arrow indicates the external coat of the esophagus.

The patients were instructed to fast for ≥24 hours and undergo EGD one or two days before the procedure to cleanse the esophagus of any residual material. The length of the LES was measured on HRM. Myotomy measuring 4 cm was used to ensure complete dissection of the LES. POEM was performed under general anesthesia with airway intubation. A forward-viewing endoscope with CO_2_ insufflation was used. A transparent plastic cap was attached to the endoscope tip. Before beginning the procedure, the gastroesophageal junction (GEJ) was identified, and its distance from the incisors was determined. The site of submucosal tunnel entry was selected as 6 cm proximal to the GEJ. The submucosal tunnel was terminated 2 cm distal to the GEJ. The GEJ was confirmed based on the distance to the incisors through the esophageal lumen (esophageal tunnel) and the identification of increased vascularity with spindle-shaped veins in the tunnel. We performed a full-thickness dissection of both the circular and longitudinal muscle layers of the LES and the cardiac sphincter (Figure 1). The full-thickness dissection length was 4 cm, each being 2 cm proximal and distal to the GEJ (equivalent to the length of the LES and the cardiac sphincter extending 1 cm on both sides). Finally, the entry was closed with hemostatic clips to avoid potential leakage of luminal fluid into the tunnel and mediastinum. All procedures were performed by expert endoscopists with 10 years of experience in endoscopic procedures at two medical centers.

### 2.4. Post-POEM

Patients were hospitalized after the procedure and administered intravenous antibiotics. After computed tomography confirmed the absence of perforation, a 24-hour fasting period was ensured before commencing clear water intake on day 1 postoperatively. On day 2, a soft diet was started and maintained for several days, followed by a regular diet.

### 2.5. Outcomes and Follow-Up

The primary outcomes of the study were the technical and clinical success rates. Technical success was defined as the successful completion of POEM. Clinical success was defined as an Eckardt score of ≤3 after POEM. The secondary outcomes included POEM-related adverse events, IRP and LESP on HRM before and after POEM, reflux-related adverse events, and procedure time. Mucosal injury, perforation, bleeding, and pneumothorax were recorded as POEM-related adverse events. Reflux-related adverse events included reflux esophagitis, esophageal acid exposure, and gastroesophageal reflux disease (GERD) symptoms. Reflux esophagitis was confirmed by EGD and classified by the Los Angeles classification [5]. A percentage of acid exposure time (%AET, esophageal pH < 4) of >4.2% was defined as abnormal acid exposure [6]. GERD symptoms were evaluated using the GERD-*Q* score, and a GERD − *Q* score > 7 was considered to indicate significant GERD symptoms [7].

The EGD, HRM, and Eckardt score evaluations for all patients were scheduled at 6 and 12 months after POEM. Twenty-four-hour pH measurements and GERD-Q questionnaire scores were evaluated at 12 months after POEM. The Eckardt score was retrieved telephonically by interviewing patients every 6 months after POEM.

### 2.6. Statistical Analysis

Continuous variables are presented as the mean ± standard deviation (SD) or median with range and were tested using paired nonparametric testing. Statistical significance was set at *P* < 0.05. The data were analyzed with IBM SPSS 19.0 software.

## 3. Results

### 3.1. Patient Information

Overall, 31 patients (median age: 45 years, range: 31-64 years; eighteen women, thirteen men) were included. The symptoms lasted for a median of 4 (range: 2.6–8.7) years. Eight patients (25.8%) received treatment for achalasia before POEM, 5 (16.1%) received botox injections, 2 (6.5%) underwent PD, and one patient (3.2%) underwent bougie dilation.  Table 1 summarizes the data of the measured outcomes.

### 3.2. POEM Details

All patients successfully underwent POEM procedures, with durations lasting 28–80 (median: 38) minutes. One patient (3.2%) experienced mucosal injury and required perioperative endoscopic clipping of the wound. No patient had perforation, severe bleeding, or pneumothorax (Table 2).

### 3.3. Efficacy of POEM

The Eckardt score, LESP, and IRP data at 6 months and 12 months after POEM procedures were obtained from 31 patients. The LESP and IRP showed a significant reduction at 6 and 12 months postoperatively. The median LESP decreased from 31.6 to 13.4 mmHg at 6 months after POEM (*P* < 0.001) and to 11.8 mmHg at 12 months after POEM (*P* < 0.001). The median IRP decreased from 27.8 to 12.9 mmHg at 6 months after POEM (*P* < 0.001) and to 11.6 mmHg at 12 months after POEM (*P* < 0.001). At the 6- and 12-month follow-up, the median Eckardt score decreased from 7 (5-10) to 0 (0-2) and 1 (0-2), respectively (Table 3). No patient had an Eckardt score > 3 at 12 months after POEM. The overall clinical success rate was 100% (31/31) within 1 year post-POEM. After 2 years, the available Eckardt scores of 23 patients (74.2%) were still ≤3. The clinical success rate was 100% (23/23). The Eckardt data collected by interview of 9 patients (29.0%) were obtained beyond 2 years post-POEM; only one patient (11.1%, 1/9) required PD because of worsening symptoms (Eckardt score = 5). The overall clinical success rate was 88.9% (8/9).

### 3.4. Reflux-Related Adverse Events

At the 12-month follow-up, a 24 h pH monitoring test and EGD were performed in all patients. Abnormal esophageal acid exposure was observed in 6 cases (19.4%). Four cases (12.9%) of esophagitis (Los Angeles classification A, 3; B, 1) were confirmed by EGD. The patients received a double dose of proton pump inhibitors (PPI) for 6 weeks, followed by repeat EGD, and their esophagitis was found to be completely resolved. Two patients (6.5%) had GERD symptoms and experienced complete symptom remission after standard-dose PPI treatment for 6 weeks (Table 2).

## 4. Discussion

Achalasia is a rare, primary motility disorder caused by decreased or lost myenteric neurons [8–10]. Patients with achalasia achieve remission due to the reduction in LES and GEJ pressure after treatment with PD, LHM, and endoscopic injection of botulinum toxin [11, 12]. However, these therapies have limitations: PD requires multiple-grade dilations to establish symptom remission, and esophageal perforation occurs frequently during the large dilatations performed initially [13]. Endoscopic injection of botulinum toxin has shown good safety; however, its effects last for only a few months [14]. LHM has traditionally been preferred for achalasia; however, physicians always additionally perform partial fundoplication to reduce the postoperative risk of GERD [15].

POEM was first performed in 2008 and reported in 2010 [3], with various reports on its safety and effectiveness [16–19]. The standard POEM involves a 7 cm myotomy and incision of the muscle layer of the circular muscle bundles [3]. However, in clinical practice, a shorter or full-thickness myotomy has been performed, which shows comparable outcomes to the standard POEM [20, 21]. However, the safety and efficacy of a combined shorter and full-thickness myotomy have not been reported. Thus, we performed this study to evaluate the safety and effectiveness of this modified myotomy technique.

In this study, shorter and full-thickness POEM was successfully performed in all patients with type II achalasia. In our study, the clinical success rates were 100%, 100%, 100%, and 88.9% within six months, 1 year, 2 years, and beyond 2 years after the procedure, respectively. Based on data from recently published literature, the clinical success rates of POEM procedures ranged from 87.9% to 100% at 1 year after POEM [21–25]. The treatment efficacy within 1 year post-POEM in the current study was comparable to that of these studies. A long-term follow-up reported that the clinical success rate was 90.3% at two years after standard POEM [26], which was lower than our results. In our study, the clinical success rate was 100%, which was maintained for 2 years. The findings demonstrated that shorter and full-thickness POEM was more effective than standard POEM. The reason may be the type II achalasia in our study, which is associated with an excellent outcome for POEM [27]. Furthermore, POEM was performed by experienced endoscopists in our study, which may be attributable to the excellent outcome of POEM.

The key point of successful POEM for achalasia was the complete incision of the LES. The mean length of the LES was 3.1 cm in this study. The myotomy length in the esophagus was set at 4 cm to ensure that the LES incision was completely performed. Furthermore, the length of the LES was measured using HRM. The maximum length of the LES was 3.8 cm in our study; thus, a 4 cm myotomy length in the esophagus was sufficient to dissect the LES completely. Recently, a study reported that a shorter POEM (the length of esophageal myotomy was ~3–4 cm) for type II achalasia showed excellent outcomes during the follow-up period [22]. Shorter POEM can avoid unnecessary esophageal muscle excision and may reduce the risk of procedure-related adverse events. Furthermore, shorter myotomy is a better option for complex achalasia, for example, in cases involving a sigmoid-type esophagus. Most endoscopists perform selective dissection of the circular muscle layer alone under the guidance of a standard POEM. However, some medical centers dissect both the circular and longitudinal muscle layers. In the POEM procedure, it is difficult to ensure the dissection of the circular muscle only. Previously, endoscopists required excessive time to protect the fragile longitudinal muscle layer if only the circular muscles were dissected during the POEM procedures. In the current study, the median procedure time was 38 min, which was less than that of a standard and shorter POEM [20, 28]. This may be because our technique involved both a shorter and full-thickness POEM, which could reduce the procedure time [20, 21].

Concerning procedure-related adverse events, only one patient (3.2%) experienced mucosal injury in our study. According to recent reports, procedure-related adverse event rates vary between 3.2% and 13.8%, which is consistent with our results [16, 21, 29]. There is a consensus that GERD is a common complication after POEM. According to recent reports, the incidence of GERD after POEM ranges from 16.8% to 57.8% [16, 30–32]. A shorter POEM may decrease the risk of subsequent GERD because the antireflux barrier in the esophagus is well preserved. A randomized controlled trial that compared standard and shorter POEM treatment for type II achalasia reported abnormal esophageal acid exposure rates in the shorter myotomy group, which were significantly lower than those in the standard group (23.9% vs. 43.8%) [22]. The incidences of GERD symptoms and reflux esophagitis were 15.2% and 8.7% in this study, respectively. Our study reported an incidence of abnormal esophageal acid exposure (19.4%), GERD symptoms (12.9%), and reflux esophagitis (6.5%), which was similar to the corresponding results from a previous study [22]. The findings demonstrated that shorter and full-thickness POEM may reduce the incidence of acid reflux-related adverse events. Full-thickness myotomy may increase the incidence of acid reflux-related adverse events after POEM. Interestingly, the incidence of acid reflux-related adverse events was not different between full-thickness myotomy and circular muscle myotomy in some reports [20, 23, 33]. Our results suggest that shorter and full-thickness myotomy did not increase the postoperative incidence of acid reflux-related adverse events. Thus, there is no need to deliberately protect the longitudinal muscles. The findings of our study demonstrated that shorter and full-thickness myotomy could decrease the procedure time and was potentially more effective than standard POEM.

There are some limitations to our study. First, this study had a small sample size; it was a retrospective and nonrandomized study, although patients from two centers were enrolled. Moreover, only type II achalasia patients were enrolled in our study, suggesting that our results may not be suitable for other types of achalasia. Hence, future research should involve a large-scale prospective randomized controlled study design, which can confirm our findings effectively.

## 5. Conclusions

In summary, a shorter and full-thickness POEM is safe and feasible for the treatment of patients with type II achalasia. Our findings suggest that this shorter and full-thickness POEM can improve the quality of life of such patients.

## Figures and Tables

**Table 1:** Patient characteristics.

	*N* = 31
Age, median (range), year	45 (31-64)
Female/male	18/13
Duration of symptoms, median (range), years	4 (2.6-8.7)
The length of LES, mean ± SD, mm	3.1 ± 0.5
Eckardt score, median (range)	7 (5-10)
LESP, median (range), mmHg	31.6 (26.7-49.7)
IRP, median (range), mmHg	27.8 (20.6-47.5)
Previous treatment, *n* (%)	
Botox injection	5 (16.1)
Pneumatic dilation	2 (6.5)
Bougie dilation	1 (3.2)
No treatment	23 (74.2)

**Table 2:** POEM outcomes.

	*N* = 31
Technology success rates, *n* (%)	31 (100)
Procedures time, median (range), min	38 (28-80)
POEM-related adverse events, *n* (%)	
Mucosal injury	1 (3.2)
Perforation	0 (0)
Severe bleeding	0 (0)
Pneumothorax	0 (0)
Reflux-related adverse events, *n* (%)	
Abnormal acid exposure, *n* (%)	6 (19.4)
Endoscopic esophagitis, *n* (%)	4 (12.9)
GERD symptom, *n* (%)	2 (6.5)
GERD-*Q* score	4.39 ± 2.45

**Table 3:** Eckardt score, LESP, and IRP data pre- and postoperative POEM.

	Pre-POEM	6 months after POEM	12 months after POEM	*P* value
Eckardt score	7 (5-10)	0 (0-2)	1 (0-2)	<0.001
LESP, mmHg	31.6 (26.7-49.7)	13.4 (10.5-21.6)	11.8 (7.4-16.7)	<0.001
IRP, mmHg	27.8 (20.6-37.5)	12.9 (11.3-23.4)	11.6 (9.6-16.8)	<0.001

## Data Availability

The datasets used or analyzed during the current study are available from the corresponding authors on reasonable request.
